# Bone morphogenetic protein 4 modulates c-Kit expression and differentiation potential in murine embryonic aorta-gonad-mesonephros haematopoiesis *in vitro*

**DOI:** 10.1111/j.1365-2141.2007.06795.x

**Published:** 2007-10-01

**Authors:** Caroline J Marshall, Joanna C Sinclair, Adrian J Thrasher, Christine Kinnon

**Affiliations:** 1 Molecular Immunology Unit, UCL Institute of Child Health London, UK; 2 Department of Clinical Immunology, Great Ormond Street Hospital NHS Trust London, UK

**Keywords:** Bone morphogenetic protein, haematopoiesis, aorta-gonad-mesonephros, c-Kit, haemangioblast

## Abstract

The transforming growth factor-*β*-related factor bone morphogenetic protein 4 (BMP4) is expressed in the human embryonic aorta-gonad-mesonephros (AGM) coincident with the emergence of haematopoietic cells and influences postnatal mammalian haematopoietic stem cells *in vitro*. To investigate the role of BMP4 in mammalian embryonic haematopoiesis, cells were isolated from murine AGM and two populations of CD34^+^ cells with different levels of c-Kit expression and multipotency were identified. CD34^+^/c-Kit^high^ cells express CD45 and are haematopoietic-restricted progenitors. In contrast, CD34^+^/c-Kit^low^ cells are Flk1+/CD45^neg^ and generate adherent colonies in *ex vivo* culture that resemble haemangioblast colonies identified in other systems. The addition of BMP4 to AGM cells resulted in expansion of the CD34^+^/c-Kit^low^ cell pool within 48 h, via a combination of down modulation of the c-Kit receptor in CD34^+^/c-Kit^high^ cells and proliferation. In long-term culture, BMP4 increased the growth/survival of CD34^+^/c-Kit^high^ haematopoietic progenitors, effects that were blocked by BMP inhibitors. CD34^+^/c-Kit^high^ progenitors cultured with BMP4 also generated adherent colonies typical of c-Kit^low^ cells. These results suggest that BMP4 regulates c-Kit expression and differentiation potential in CD34^+^ AGM cells and supports a role for BMP signalling in the maintenance of multipotency during embryonic haematopoiesis, providing an insight into stem cell homeostasis within the mammalian haematopoietic niche.

Haematopoietic stem cells (HSCs) capable of long-term multilineage reconstitution in myeloablated recipient mice first arise during embryogenesis and persist into adult life. The differentiation pathways and cytokine requirements through which haematopoietic progenitor cells achieve maturity in all blood cell lineages have been studied extensively and are well characterized. However, the processes by which the stem cells and precursors that seed the adult multilineage blood system are generated and maintained are less well understood. Stem cell homeostasis depends on maintaining a balance between self-renewal, proliferation, death and differentiation. Identification of the factors that regulate HSC emergence and subsequent development will provide insight into how the mammalian haematopoietic system is formed and how the decision to self-renew or differentiate is controlled at the molecular level.

During mammalian embryogenesis, the first multipotent HSCs are detected within a region of mesodermal tissue containing the dorsal aorta, gonadal ridge and mesonephros, the aorta-gonad-mesonephros (AGM) region, at between 8·5 and 11·5 day post coitum (dpc) in the mouse and 4–6 weeks gestation in the human embryo (Godin *et al*, 1995; Medvinsky & Dzierzak, 1996; Godin *et al*, 1999; Tavian *et al*, 2001). Within this region, CD45^+^ haematopoietic cells aggregate in clusters associated with major vessel walls, including the ventral wall of the dorsal aorta (Tavian *et al*, 1996). However, the precise origins of this specific population of embryonic cells and the nature of the signals regulating their emergence and subsequent development have yet to be fully defined.

Progenitors that display haemangioblast (haematopoietic and endothelial) potential *in vitro*, similar to embryonic stem (ES) cell lines, have been identified early in the development of the gastrulating mouse embryo (Choi *et al*, 1998; Huber *et al*, 2004). The proximity of intraaortic HSCs to endothelial cells lining the dorsal aorta, combined with the overlapping expression profiles between the two populations, suggest that HSCs and endothelial cells arise from a bipotential precursor, the haemangioblast, located within or adjacent to the aortic wall (Marshall & Thrasher, 2001). In support of this, CD34^+^/CD45^−^ endothelial cells isolated from 5-week human AGM lack haematopoietic potential initially but produce blood cells when cultured in the presence of stromal cells that support multilineage haematopoiesis (Oberlin *et al*, 2002). Haematopoietic output from endothelial cells both quantitatively and temporally reflects the degree of haematopoietic activity *in vivo* and is restricted to blood-forming tissues.

A number of extrinsic factors have been reported to influence haematopoiesis including members of the hedgehog, Wnt, Notch and transforming growth factor-*β* (TGF*β*)/bone morphogenetic protein (BMP) families (Fortunel *et al*, 2000; Bhardwaj *et al*, 2001; Kumano *et al*, 2003; Reya *et al*, 2003; Sadlon *et al*, 2004; Androutsellis-Theotokis *et al*, 2006; Neves *et al*, 2006). BMPs have a broad spectrum of effects during embryogenesis, ranging from induction of ventral mesoderm to patterning a variety of tissues (Winnier *et al*, 1995; Hogan, 1996). During lower vertebrate (*Xenopus*) embryogenesis the genetic programming of putative haemangioblasts is regulated by bone morphogenetic protein 4 (BMP4) (Walmsley *et al*, 2002). BMPs also enhance self-renewal and increase the haematopoietic potential of ES cells (Johansson & Wiles, 1995; Chadwick *et al*, 2003; Chambers & Smith, 2004). Recently, BMP signalling has been implicated in adult murine bone marrow homeostasis in the regulation of niche size and stem cell numbers (Zhang *et al*, 2003). *In vitro*, while BMP4 maintains the proliferative potential of cord blood-derived CD34^+^ haematopoietic progenitors, TGF*β* regulates cell cycle status and expression of the stem cell factor receptor c-Kit to maintain a primitive, undifferentiated population (Sansilvestri *et al*, 1995; Bhatia *et al*, 1999; Morita *et al*, 2003).

We have previously shown that BMP4 is expressed in a distinctive pattern underlying the ventral wall of the dorsal aorta associated with the appearance of intraaortic haematopoietic clusters in the AGM region of 5-week old human embryos (Marshall *et al*, 2000). To investigate the role of BMP4 at the onset of multipotent haematopoietic development during mammalian embryogenesis, we have isolated cells co-expressing CD34 and c-Kit from the AGM region of mid-gestation murine embryos (9·5–11·5 dpc), at the time of normal HSC emergence. This CD34^+^/c-Kit+ population has previously been shown to give rise to cells of all haematopoietic lineages *in vitro* indicating that it contains multipotent HSCs (Delassus *et al*, 1999). Coincident with the peak of AGM haematopoietic activity *in vivo*, we have identified two populations of CD34^+^ AGM cells that differ in c-Kit expression and differentiation potential. BMP4, specifically, modulates the size and potential of these populations *ex vivo*, similar to the effects of TGF*β* in the adult blood system. This effect can be neutralized by inhibition of BMP signalling using antagonists. These findings, together with the observed *in vivo* expression pattern, support a role for BMP4 in the development and regulation of early haematopoietic progenitors within the mammalian embryonic AGM region.

## Methods

### AGM dissection and cell preparation

Timed matings of wild-type CD1 mice generated embryos at embryonic day (9·5, 10·5 and 11·5). From each embryo, the AGM region between the anterior limb bud and umbilical vessels, containing the dorsal aorta, was dissected in phosphate-buffered saline (PBS). AGMs from single litters were pooled and dissociated in cell dissociation buffer (Invitrogen Ltd, Paisley, UK) for 15 min at 37°C followed by gentle trituration through 23 and 25 G needles. Single cells were filtered through a 70 *μ*m cell strainer and washed with Hank's balanced salt solution (HBSS) supplemented with 1% bovine serum albumin (BSA).

### Flow cytometry and fluorescence-activated cell sorting

Dissociated AGM cells were incubated with fluorescence-conjugated antibodies (BD Biosciences Pharmingen, San Diego, CA, USA) for 30 min at 4°C. Antibodies used for flow cytometry were: rat anti-mouse CD34 (RAM34)-fluorescein isothiocyanate (FITC) or phycoerythrin (PE); Flk1 (Avas12*α*1)-PE; CD117 (c-Kit/2B8)-allophycocyanin (APC) and CD45(30-F11)-peridinin chlorophyll (PerCP). Antibodies for fluorescence-activated cell sorting (FACS) were: rat anti-mouse CD34 (RAM34)-FITC and CD117 (c-Kit/2B8)-PE. Cells were washed with HBSS/1% BSA and either sorted using an EPICS Altra fluorescent activated cell sorter with Autoclone® sorting option (Beckman Coulter, High Wycombe, UK) or analysed using a CyAn ADP flow cytometer (Dakocytomation, Cambridge, UK).

### Cell culture

Total AGM cells were plated into 6-well tissue culture plates. FACS-sorted cells were dispensed directly into 96-well tissue culture plates at 50 cells/well onto adherent irradiated S17 stromal cells (plated at 2000 cells/well 24 h previously). Cells were cultured in serum-free CellGro stem cell growth medium (CellGenix, Freiburg, Germany) supplemented with 10^−4^mol/l 2-mercaptoethanol. Additional factors were added as appropriate on day 1, including recombinant human BMP4 (10 ng/ml), murine BMPR-IB/ALK-6/Fc chimaera (200 ng/ml) and murine Noggin/Fc chimaera (200 ng/ml) (all R&D Systems, Minneapolis, MN, USA), 25 ng/ml stem cell factor (SCF) (Peprotech Inc, Rocky Hill, NJ, USA). Human BMP4 has been shown to be biochemically active in the murine carcinoma-derived chondrogenic cell line ATCD5. Cells were incubated for 2, 10 or 20 d at 37°C, 5% CO_2_. To avoid disturbing the small number of cells plated, the medium was not changed during the 2- and 10-d culture periods. For 20-d cultures, medium was changed at day 10 and BMP4 re-added. Statistical analyses were performed using the Wilcoxon signed ranks test (same embryonic age) and the Kruskal–Wallis test (different embryonic ages). For flow cytometric analysis, cells were labelled with the fluorescent dye tetramethyl-6-carboxyrhodamine [TAMRA; 1 ng/ml in minimum essential medium (MEM)/PBS] at 37°C for 15 min.

### Colony-forming unit assay

Primary colonies of AGM-derived cells were trypsinised, washed and resuspended to a single-cell suspension in Iscove's Modified Dulbeccos medium (Invitrogen Ltd). Disrupted colonies were plated into 35 mm bacterial Petri dishes in Methocult GF complete methylcellulose medium containing recombinant cytokines [erythropoietin, interleukin (IL)-3, IL-6, stem cell factor (SCF)] suitable for colony assays of murine cells (Stemcell Technologies Inc., Vancouver, BC, Canada). Colonies were scored by morphology after 20 d.

### Microscopy

Colonies/cells were analysed using a Nikon Eclipse TS100 inverted microscope (Nikon Corporation, Tokyo, Japan) with a 20×/0·40 objective for bright field. Images were captured with a Nikon Coolpix 4500 digital camera and processed using Adobe Photoshop 5·5 (Adobe systems, San Jose, CA, USA).

## Results

### Mid-gestation murine CD34^+^ AGM cells generate morphologically different colonies depending on level of c-Kit expression

**Fig 1:**
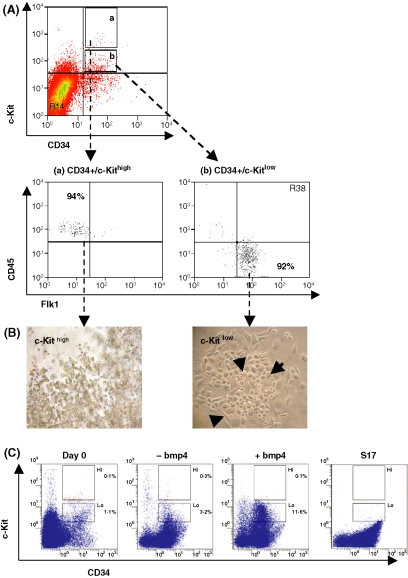
Mid gestation murine CD34^+^ aorta-gonad-mesonephros (AGM) cells contain subpopulations that differ in levels of c-Kit expression, differentiation potential and response to bone morphogenetic protein 4 (BMP4). (A) AGM cells (10·5 dpc) were analysed by flow cytometry (CyAn ADP Flow Cytometer) for expression of CD34, c-Kit, Flk1 and CD45 to identify subpopulations. Representative results are shown. CD34^+^/c-Kit+ cells can be subdivided into c-Kit^high^ and c-Kit^low^ fractions *(areas a & b)*. Differential expression profiles of *(a)* CD34^+^/c-Kit^high^ and *(b)* CD34^+^/c-Kit^low^ fractions for expression of Flk1 and CD45. Dotted arrows indicate expansion of gated areas *a* and *b* as indicated. To maintain accuracy, analysis of the c-Kit^low^ and c-Kit^high^ cell fractions could only be performed on small numbers of cells per experiment (pooled littermates) however the distribution of expression was consistent between experiments. (B) CD34^+^/c-Kit^high^ cells generate haematopoietic colonies of typical CFU-GM morphology. CD34^+^/c-Kit^low^ cells generate exclusively adherent colonies containing multiple cell types: phase-dim spindle-shaped cells (lower arrowhead), large round cells (arrow) and phase-bright small, round cells (upper arrowhead). (C) Unsorted (total) AGM cells were cultured in serum-free conditions −/+ recombinant BMP4 (10 ng/ml) for 2 d and changes in the CD34^+^/c-Kit^high/low^ subpopulations analysed by flow cytometry (CyAn ADP cytometer). Without added BMP4 (−BMP4), the percentage of CD34^+^/c-Kit^high/low^ cells increases slightly in culture from day 0 but the ratio of CD34^+^/c-Kit^high^ to CD34^+^/c-Kit^low^ remains relatively constant. With the addition of BMP4, there is a considerable increase in the CD34^+^/c-Kit^low^ population compared to day 0 and to cells cultured without added BMP4. Irradiated S17 feeder cells appear to express CD34, accounting for the apparent increase in CD34^+^/c-Kit^neg^ in cultured cells, but are c-Kit^neg^.

Flow cytometric analysis of cells isolated from mid-gestation murine embryos (pooled 10·5 dpc littermates) suggested that the AGM contained two subpopulations of CD34^+^ cells that differentially expressed c-Kit (Fig 1A). These two subpopulations were also analysed for expression of the vascular endothelial growth factor receptor Flk1 and the pan-leucocyte marker CD45. The majority of CD34^+^/c-Kit^high^ cells were Flk1^neg^/CD45^+^ and probably correspond to cells within the intraaortic haematopoietic clusters. In contrast, CD34^+^/c-Kit^low^ cells were mainly Flk1+/CD45^neg^.

To assess haematopoietic potential, FACS-sorted AGM cells were plated in methylcellulose medium containing a cocktail of cytokines to identify colony-forming cells. All colony-forming unit (CFU) activity was contained within the CD34^+^/c-Kit+ positive population but the potential of colony-forming cells differed depending on the level of c-Kit expression (Fig 1B). Haematopoietic granulocyte-macrophage CFU (CFU-GM) activity was restricted to the CD34^+^/c-Kit^high^ cell fraction with a frequency of 2000 CFU per 1 × 10^6^ CD34^+^/c-Kit^high^ cells. In contrast, CD34^+^/c-Kit^low^ cells generated exclusively adherent colonies containing a combination of three morphologically distinct cell types: phase-dim spindle-shaped cells; large round cells and clusters of small, round phase-bright cells. Cells that did not express either marker (CD34^−^/c-Kit^neg^) failed to generate any colonies. Similarly, no CFU activity was detectable in the single-positive cell fractions (CD34^+^/c-Kit^neg^, CD34^−^/c-Kit+).

We investigated the effect of BMP4 on c-Kit expression levels in AGM-derived murine cells. Unsorted AGM cells (10·5 dpc) were cultured on a low-density monolayer of irradiated S17 stromal cells in serum-free medium alone or medium supplemented with recombinant BMP4 (10 ng/ml). Cells were collected at day 2 and analysed by flow cytometry for CD34 and c-Kit expression compared to the starting population. In a representative experiment, CD34^+^/c-Kit^high^ and CD34^+^/c-Kit^low^ cells initially comprised around 0·1% and 1% of total AGM cells respectively (Fig 1C). After 2 d in serum-free culture (−BMP4) there was a minimal increase in CD34^+^/c-Kit+ cells although the ratio of CD34^+^/c-Kit^high^ and CD34^+^/c-Kit^low^ cells was similar to day 0. In contrast, addition of BMP4 (+BMP4) resulted in a greater expansion in the CD34^+^/c-Kit^low^ population (from 3·2% to 11·6%) compared to day 0 and serum-free controls.

### BMP4 increases the growth/survival of AGM-derived CD34^+^/c-Kit^high^ cells *in vitro*

Modulation of c-Kit expression may be one mechanism by which the balance between blood cell precursors and progenitors is maintained during AGM haematopoiesis. We therefore investigated the longer term effects of BMP4 on AGM-derived CD34^+^/c-Kit^high^ cells isolated by FACS from embryos aged between 9·5 and 11·5 dpc and cultured as above on irradiated S17 stromal cells in serum-free conditions with or without BMP4 (10 ng/ml) for 10 d. At the end of the culture period, the number of AGM-derived colonies generated under each condition (plating efficiency) was scored.

**Fig 2:**
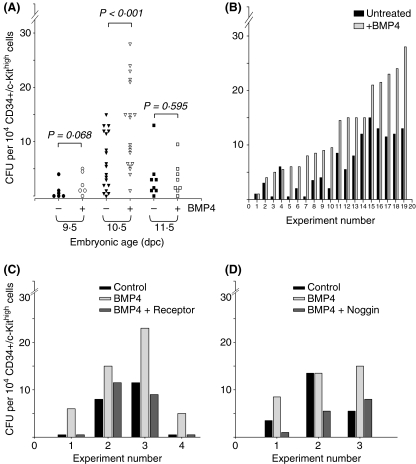
Bone morphogenetic protein 4 (BMP4) increases the plating efficiency (growth/survival) of mid-gestation aorta-gonad-mesonephros (AGM)-derived CD34^+^/c-Kit^high^ cells in 10-d culture. (A) Addition of BMP4 (open symbols) results in an increase in the number of colonies produced at 10·5 dpc compared with serum-free conditions (−BMP4, solid symbols). Statistical analyses were performed using the Kruskal–Wallis test (different embryonic ages) and the non-parametric Wilcoxon signed rank test to compare the collective results for a given embryonic age with and without addition of BMP4. Results were found to be significant at 10·5 dpc (*P* < 0·001) but not significant at 9·5 dpc or 11·5 dpc (*P* = 0·068 and *P* = 0·595 respectively). (B) The effect of BMP4 was consistent at 10·5 dpc, increasing plating efficiency/CFUs by 50% or greater in 12 out of 19 separate experiments. BMP inhibitors. (C) soluble BMP type I-receptor (200 ng/ml) and (D) Noggin (200 ng/ml) neutralize the effect of BMP4 on cultured CD34^+^/c-Kit^high^ cells (10·5 dpc).

The plating efficiency of CD34^+^/c-Kit^high^ cells isolated from 9·5 dpc and 11·5 dpc AGM was generally low (<10 CFU/10^4^ cells plated) compared with 10·5 dpc (up to 30 CFU/10^4^ cells), suggesting that *ex vivo* survival or growth capacity of CD34^+^/c-Kit^high^ cells differed with embryonic age. Addition of BMP4 to 9·5 or 11·5 dpc CD34^+^/c-Kit^high^ cells resulted in only minimal increases in plating efficiency compared to medium only controls (Fig 2A). In contrast, the number of colonies generated from BMP4-treated 10·5 dpc CD34^+^/c-Kit^high^ cells increased significantly (*P* < 0·001) and reproducibly with a greater than twofold increase in 14 out of 19 independent experiments, some reaching as high as 16 times control levels (Fig 2B). Addition of c-Kit ligand (25 ng/ml), either alone or in combination with BMP4, had no observable effect on colony number, size or morphology.

### The effects of BMP4 are neutralized by BMP-antagonists

To test whether the observed increase in colony formation from 10·5 dpc AGM-derived CD34^+^/c-Kit^high^ cells was BMP-specific, the high-affinity BMP-binding protein noggin and recombinant soluble BMP type I-receptor (BMPR-IB) were added in combination with BMP4 to block BMP signalling in culture. Noggin is one of a number of antagonistic agents that regulate BMP bioactivity during embryonic development by sequestering BMP molecules in the extracellular space. Recombinant BMPR acts in a similar way *in vitro*, binding BMP4 with high affinity in solution. After 10 d in culture with BMPR or noggin (200 ng/ml) the number of colonies generated fell to control levels or less (Fig 2C and D). The inhibitors were not toxic at the concentrations used and the number, size and morphology of colonies were comparable to untreated controls (−BMP4). In one experiment (Fig 2D, experiment 2), although BMP4 alone had no significant effect, addition of noggin reduced the number of colonies formed. However, this result was not typical and in other experiments noggin had a similar effect to BMP-R.

The ability of AGM-derived CD34^+^/c-Kit^high^ cells to survive and/or proliferate *in vitro*, therefore, increased in the presence of exogenous BMP4. This effect was blocked by BMP inhibitors and, at this developmental stage, did not require the addition of c-Kit ligand.

### BMP4-treated CD34^+^/c-Kit^high^ AGM cells generate adherent colonies in CFU-assays similar to CD34^+^/c-Kit^low^ cells

The level of c-Kit expression was an indicator of the differentiation potential of 10·5 dpc CD34^+^ AGM cells (Fig 1B). In methylcellulose assays, isolated CD34^+^/c-Kit^high^ cells generated haematopoietic colonies typical of committed progenitors. To assess the effect of BMP4 on the colony-forming potential of committed progenitors, colonies generated in 10- or 20-d cultures from FACS-isolated CD34^+^/c-Kit^high^ cells were plated into methylcellulose containing a combination of haematopoietic cytokines for 2–3 weeks. The small number of cells per colony allowed very low density distribution in methylcellulose, however, the exact number of cells plated could not be accurately counted therefore quantitative comparison was not attempted.

**Fig 3:**
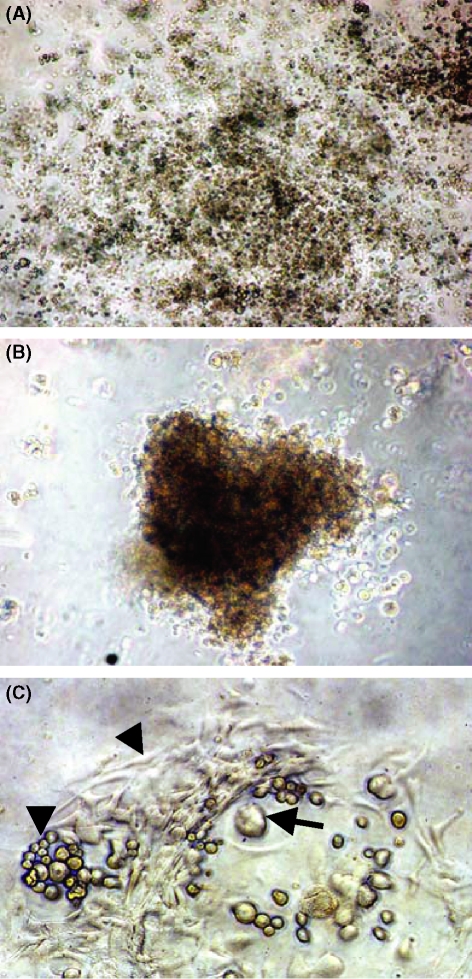
CD34^+^/c-Kit^high^ aorta-gonad-mesonephros (AGM) cells cultured with bone morphogenetic protein 4 (BMP4) generate adherent colonies resembling CD34^+^/c-Kit^low^ colonies. (A) CD34^+^/c-Kit^high^ cells isolated by FACS from AGM tissue (10·5 dpc) and cultured for 10 d in serum-free medium alone (−BMP4) generate haematopoietic colonies composed of granulocytic/myeloid cells (CFU-GM). BMP4-treated CD34^+^/c-Kit^high^ cells also produce (B) haematopoietic colonies which also contain erythroid cells (CFU-GEMM) and (C) at a similar frequency, adherent colonies which resemble colonies generated from freshly isolated CD34^+^/c-Kit^low^ cells of the same embryonic age. Adherent colonies contain three morphologically distinct cell types: phase-dim spindle-shaped cells (arrowhead), large round cells (arrow) and phase-bright small, round cells (hatched arrow).

CD34^+^/c-Kit^high^ AGM cells cultured for 10 d or more in serum-free medium alone or with BMP4 retained the capacity to generate granulocyte/monocyte colonies across all embryonic ages tested (Table I, Fig 3A), suggesting that lineage-restricted haematopoietic progenitors (CFU-GM) were maintained or generated within the cultures. BMP4-treated CD34^+^/c-Kit^high^ cells also generated colonies that contained erythroid cells, indicating the presence of more primitive, less restricted progenitors (CFU-GEMM) (Fig 3B).

**Table I:** Morphology of colonies derived from CD34^+^/c-Kit^high^ aorta-gonad-mesonephros (AGM) cells pre-cultured −/+ bone morphogenetic protein 4 (BMP4) at different embryonic ages. Numbers are expressed as the percentage of AGM-derived colony-forming units/primary colonies that produced non-adherent haematopoietic and/or adherent colonies in secondary methylcellulose culture. Adherent colonies contained a combination of three morphologically distinct cell types. Only cells pre-cultured with BMP4 produced polymorphic colonies.

		Haematopoietic colonies (%)		Adherent colonies (%)		
Age (dpc)	Time in culture (d)	−BMP4	+BMP4	−BMP4	+BMP4	*n* ± BMP4
9·5	10	100	88	0	70	7/17
10·5	10	50	83	0	66	227/30
	20	63	81	0	81	11/11
11·5	10	70	64	0	36	10/11
	20	0	30	0	0	1/10

*n*, number of primary colonies −/+ BMP4 plated in methylcellulose; dpc, days post-coitum.

Most strikingly, CD34^+^/c-Kit^high^ cells at all embryonic ages pre-cultured with BMP4 generated adherent colonies that closely resembled the colonies produced from CD34^+^/c-Kit^low^ cells at day 0 (Table I, Fig 3C). These colonies contained a mixture of adherent spindle-shaped and large round cells overlaid by clusters of smaller round cells and frequently formed extensive networks that persisted in culture for more than 4 weeks. Immunocytochemical analysis of adherent colonies showed expression of the stromal molecule STRO1 in large round cells, endothelial (Flk1, endoglin) markers in large round and spindle cells and the haematopoietic marker CD45 in small round cells. Adherent colonies therefore appeared to comprise a combination of stromal, endothelial and haematopoietic cells. In most cultures, both non-adherent haematopoietic and adherent colonies were generated, however, a number of cultures produced adherent colonies exclusively. This adherent colony-forming potential was retained by cells isolated at 10·5 dpc but lost on extended culture of 11·5 dpc cells. Importantly, no adherent colonies were generated from CD34^+^/c-Kit^high^ cultured in the absence of exogenous BMP4. The colony-forming potential of AGM-derived CD34^+^/c-Kit^high^ cells (10·5 dpc) that had been pre-cultured with soluble BMP-receptor or noggin in combination with BMP4 was also assessed (Table II). The addition of noggin prevented polymorphic colony formation although some hematopoietic (CFU-GM) colonies did develop. Soluble BMP-receptor completely blocked colony formation.

**Table II:** BMP inhibitors block formation of adherent colonies from CD34^+^/c-Kit^high^ AGM cells (10·5 dpc) pre-cultured + BMP4.

	Haematopoietic colonies (%)	Adherent colonies (%)	n
−BMP4	64	0	11
+BMP4	68	27	22
+BMP4 + soluble BMPR	0	0	5
+BMP4 + noggin	50	0	6

*n*, number of primary colonies plated in methylcellulose.

## Discussion

Stem cells play an essential role in the generation (embryonic/foetal) and regeneration (adult) of tissues. To fulfil these roles, stem cells must survive and persist within tissues and also be able to generate specialized, differentiated progeny as required. Maintenance of the stem cell pool therefore requires a precise balance between a number of processes: self-renewal (quiescence), proliferation, differentiation and apoptosis. These processes are regulated by a complex network of environmental (niche) signals linked to intrinsic pathways that can have either a permissive or an inhibitory effect on stem cell behaviour. BMP is an example of an extracellular molecule that functions in this way, with distinct or overlapping functions that induce or inhibit, depending on the stem cell system and specificity of receptor binding (Zhang & Li, 2005).

The mammalian AGM region contains a niche in which haematopoietic stem cells are generated from a pre-haematopoietic cell during embryogenesis and BMP4 is expressed during this period of HSC emergence *in vivo* in human embryos (Marshall *et al*, 2000). The AGM is possibly the only intraembryonic site of multipotent HSC specification during development, with subsequent foetal liver and bone marrow haematopoiesis seeded by the self-renewal and proliferation of AGM-derived cells rather than the *de novo* generation of HSCs in these tissues. The AGM therefore provides an important and possibly unique environment for the study of early haematopoietic ontogeny in terms of cellular origin and molecular regulation.

The most primitive haematopoietic progenitors in human bone marrow express the stem cell factor receptor c-Kit at low levels compared to more committed progenitors (Gunji *et al*, 1993; Katayama *et al*, 1993; Doi *et al*, 1997; Lian *et al*, 1997). This study showed that c-Kit expression in murine embryonic CD34^+^ AGM cells also distinguishes two subpopulations that differ in colony-forming potential. The c-Kit^high^ compartment expressed the pan-leucocyte marker CD45 and contained lineage-restricted haematopoietic progenitors (CFU-GM). In contrast, the CD34^+^/c-Kit^low^ subpopulation was CD45-negative and did not contain CFU-GM but generated adherent colonies containing multiple cell types.

CD34^+^/c-Kit^low^ AGM cells also express the vascular endothelial growth factor (VEGF) receptor Flk1. Flk1 is expressed on cell blast-colony forming cells (BL-CFCs) derived from murine ES cells in culture (Faloon *et al*, 2000). These BL-CFCs display both endothelial and haematopoietic potential and are thought to represent the *in vitro* equivalent of the haemangioblast (Choi *et al*, 1998). Flk1 expression is lost in BL-CFC-derived cells that commit to haematopoiesis. In agreement with this, AGM-derived CD34^+^/c-Kit ^high^ cells were haematopoietic and Flk1^neg^.

When CD34^+^/c-Kit+ cells were cultured with BMP4, the number of c-Kit^low^ cells rapidly increased. Tracking of c-Kit expression in *ex vivo* culture suggests that the receptor was down-modulated in CD34^+^/c-Kit^high^ cells (10·5 dpc) within 48 h (data not shown). However, the small numbers of c-Kit^high^ cells did not account for the comparatively large increase in the number of c-Kit^low^ cells. Instead, expansion of the CD34^+^/c-Kit^low^ pool appeared to result from a combination of cell division in both high and low compartments coupled with down modulation of c-Kit receptor expression in c-Kit^high^ cells (data not shown). Consistent with this, in longer-term culture with BMP4, CD34^+^/c-Kit^high^ cells had increased growth/survival and acquired similar differentiation potential to c-Kit^low^ cells in CFU assays. This effect was BMP specific and could be blocked by BMP antagonists. Comparison of different mid-gestation embryonic ages also suggested that this capacity was most evident in cells isolated from 10·5 dpc murine embryos, corresponding to the height of AGM haematopoietic activity *in vivo*.

Adherent colonies generated from freshly isolated CD34^+^/c-Kit^low^ and BMP4-treated CD34^+^/c-Kit^high^ cells contained multiple cell types and could persist and expand in culture for several weeks. Preliminary analysis of these colonies suggested that they contained a mixture of cells that express stromal, endothelial or haematopoietic markers (data not shown). They also morphologically resembled haemangioblast colonies obtained from human embryonic stem cells cultured with BMP4 (Kennedy *et al*, 2007).

In the presence of BMP4, therefore, CD34/c-Kit AGM cells underwent a change in differentiation potential coincident with a change in c-Kit expression. This concurs with the *in vivo* expression pattern of c-Kit within the embryonic AGM. In the human embryo, *KIT* mRNA is expressed at higher levels in cells within intraaortic CD45^+^ haematopoietic clusters compared to the surrounding vasculature (Labastie *et al*, 1998). AGM-derived c-Kit^high^ cells may therefore correspond to cells within intraaortic haematopoietic clusters in the mid-gestation murine AGM. Similarly, *in vivo* CD34 expression patterns in mouse and human embryos suggest that CD34^+^/c-Kit^low^ cells are located within the vascular walls including the dorsal aorta (Tavian *et al*, 1996; Wood *et al*, 1997). CD34^+^/c-Kit^low^ cells may therefore represent a precursor of or intermediate stage between endothelial and haematopoietic cells.

Our findings regarding the regulation of c-Kit expression during AGM haematopoiesis agree with and add to a recent study in which E11.5 AGM cells were cultured for a few days with fetal calf serum (FCS), SCF, basic fibroblast growth factor (bFGF) and oncostatin M (Nobuhisa *et al*, 2007). Three populations of cells were identified based on levels of CD45/c-Kit expression: CD45^low^/c-Kit+ with haematopoietic CFU activity, CD45^low^/c-Kit^neg^ granulocytes and CD45^high^/c-Kit^low/neg^ macrophages. In subsequent co-culture with fresh AGM cells, only CD45^low^/c-Kit+ cells had the capacity to reproduce, differentiate into CD45^low^/c-Kit^neg^ granulocytes and CD45^high^/c-Kit^low/neg^ macrophages and repopulate irradiated recipients, suggesting that they include HSCs. It was also observed that bFGF and oncostatin M were not required and insufficient to maintain the CD45^low^/c-Kit+ population in culture. The present study further subdivided the c-Kit+ AGM population and cultured these cells in the absence of FCS. BMP4 specifically was able to maintain the more primitive CD34^+^/c-Kit^low^/CD45^low/neg^ population. Collectively, these observations suggest that c-Kit expression during HSC development proceeds from low to high but is lost in committed haematopoietic progenitors.

Based on these results, we propose a model for mammalian embryonic haematopoietic development that requires BMP signalling at an early stage for maintenance of multipotent cells in the AGM. BMP4 maintains and possibly activates progenitors or stem cells within the ventral floor of the endothelium lining the dorsal aorta. At this point, these cells have the potential to self-renew or to differentiate along one of at least two lineage pathways. In response to BMP4 and/or other factors within the AGM niche, these progenitors proliferate and are pushed outwards from the vessel wall to form clusters in the lumen. These clusters are continuous with and connected to the vessel wall by cell–cell junctions (Marshall & Thrasher, 2001). As cells within the developing clusters move away from the aortic wall and the source of BMP, they lose multipotency and become committed to a haematopoietic fate. Exposure of lineage-committed progenitors to BMP4 *in vitro* restores a more primitive phenotype, suggesting that BMP4 maintains precursors or HSCs within the AGM, partly by blocking further haematopoietic differentiation. In the embryonic AGM, therefore, BMP4 may have a similar effect on cell behaviour to that of TGF*β* on postnatal cells in the maintenance of a primitive, multipotent population that resides within the CD34^+^/c-Kit^low^ compartment.

This model for BMP function is supported by studies on the *drosophila* ovariole in which BMP signals produced from niche cells maintain self-renewing germline stem cells by blocking differentiation (Kai & Spradling, 2004; Song *et al*, 2004). Short-range BMP-signalling induces elevated levels of a group of proteins in stem cells adjacent to cap cells at the niche interface. These proteins bind to the silencer element of a differentiation-promoting gene *bag of marbles (bam)* repressing its transcription and blocking differentiation to the next stage of germ cell development. As cells move away from the cap cells and beyond BMP signalling range, bam is expressed and the cells can differentiate. BMP also expands the BL-CFC haemangioblast population and consequently increases haematopoietic output in murine ES cells (Zafonte *et al*, 2007).

Members of the Runx family of transcription factors have also been linked to the BMP pathway. Runx1 is transiently expressed in the floor of the dorsal aorta during the period of haematopoietic activity and is required for HSC generation (North *et al*, 1999). Recently it has been demonstrated that the BMP4/Smad signalling pathway, the SCL/Tal1 transcription network and regulation of Runx1 activity are linked and determine HSC development (Pimanda *et al*, 2007). Within the embryonic AGM, BMP-activated Smad-Runx complexes may regulate expression of target genes involved in lineage commitment and haematopoietic specification.

The development of more reliable and efficient culture systems for clinical applications requires a greater knowledge of how stem cells are regulated at the molecular level. The microenvironment that supports mammalian HSC generation *in vivo* contains a variety of factors that may work synergistically or antagonistically at different stages of HSC induction, maintenance and maturation. Within the embryonic context, we have placed BMP4 within this complex network at an early stage, possibly in the regulation of haemangioblast development, resulting in the production of a pool HSCs to seed the adult haematopoietic system. Further investigations will reveal how growth factors co-operate to regulate haematopoietic development from embryo to adult and so unravel the complicated pathway that leads from mesodermal cell to blood.
